# Polypyrrole-Assisted Ag Doping Strategy to Boost Co(OH)_2_ Nanosheets on Ni Foam as a Novel Electrode for High-Performance Hybrid Supercapacitors

**DOI:** 10.3390/nano12223982

**Published:** 2022-11-11

**Authors:** Hammad Mueen Arbi, Anuja A. Yadav, Yedluri Anil Kumar, Md Moniruzzaman, Salem Alzahmi, Ihab M. Obaidat

**Affiliations:** 1 Department of Physics, United Arab Emirates University, Al Ain 15551, United Arab Emirates; 2 Department of Automotive Engineering, Yeungnam University, 280 Daehak-ro, Gyeongsan 38541, Gyeongbuk, Korea; 3 National Water and Energy Center, United Arab Emirates University, Al Ain 15551, United Arab Emirates; 4 Department of Chemical and Biological Engineering, Gachon University, 1342 Seongnam-daero, Seongnam-si 13120, Gyeonggi-do, Korea; 5 Department of Chemical & Petroleum Engineering, United Arab Emirates University, Al Ain 15551, United Arab Emirates

**Keywords:** Co(OH)_2_, Ag nanoparticle, electrode materials, hybrid supercapacitor, energy storage performance

## Abstract

Battery-type electrode materials have attracted much attention as efficient and unique types of materials for hybrid battery supercapacitors due to their multiple redox states and excellent electrical conductivity. Designing composites with high chemical and electrochemical stabilities is beneficial for improving the energy storage capability of battery-type electrode materials. We report on an interfacial engineering strategy to improve the energy storage performance of a Co(OH)_2_-based battery-type material by constructing polypyrrole-assisted and Ag-doped (Ag-doped@Co(OH)_2_@polypyrrole) nanosheets (NSs) on a Ni foam using a hydrothermal process that provides richer electroactive sites, efficient charge transportation, and an excellent mechanical stability. Physical characterization results revealed that the subsequent decoration of Ag nanoparticles on Co(OH)_2_ nanoparticles offered an efficient electrical conductivity as well as a reduced interface adsorption energy of OH^-^ in Co(OH)_2_ nanoparticles as compared to Co(OH)_2_@polypyrrole-assisted nanoparticles without Ag particles. The heterogeneous interface of the Ag-doped@Co(OH)_2_@polypyrrole composite exhibited a high specific capacity of 291.2 mAh g^−1^ at a current density of 2 A g^−1^, and showed a good cycling stability after 5000 cycles at 5 A g^−1^. The specific capacity of the doped electrode was enhanced approximately two-fold compared to that of the pure electrode. Thus, the fabricated Ag-doped@Co(OH)_2_@polypyrrole nanostructured electrodes can be a potential candidate for fabricating low-cost and high-performance energy storage supercapacitor devices.

## 1. Introduction

With the ever-increasing demand for wearable electrical and portable devices in the current world, owing to the increasing population, sustainable energy storage contributors have notably led the enhancement in ultraenergy performance hybrid energy storage applications [1,2]. As we all know, the electrochemical activities of hybrid supercapacitors depend on the utilization of battery-type electrode materials (BTEMs) [3,4,5,6,7,8,9,10,11]. However, they are still limited by their authentic lower capacities and rate performances, combined with a weaker cycling stability. Thus, it is important to improve the performance of BTEMs for hybrid supercapacitors to enhance their chances to be included in featured energy-storing applications.

Transition metal hydroxides (TMHOs) have been investigated as BTEMs for hybrid supercapacitors because of their larger theoretical capacities, excellent hydrophilicity, and simple synthesis procedures [12,13,14,15,16]. Among them, different cobalt hydroxide (Co(OH)_2_) nanoarchitectures, including nanorods [17], nanowires [18], nanoneedles [18], nanosheets [19], etc., have been largely described as optimistic BTEM candidates because of their layering construction with an ultraspace interlayer, good quality electrochemical reactions, cheaper costs, and ecofriendly benign characteristics. However, they are still limited by their dissatisfying electrochemical storing performance, including capacities that decompose at a high current density. Their lower electrical conductivities and inactive electrochemical activities, therefore, limit their large-scaling device application in hybrid supercapacitors.

Recent reports have displayed that combining TMHO materials with conducting polyaniline (PANI) (e.g., polymers (CPs) [20,21] and polypyrrole (PPy) [22,23,24]) to design CP/TMHO nanosheets (NSs) has been considered to be a practicable approach to improve their charge storing capacities. As NSs can supply excellent electrical kinetics and, thereby, faster charging transportation and electrochemical faradaic reactions, they also conserve reasonable structural stabilities, thus, enhancing the cycle span. For example, Jang et al. synthesized polypyrrole-nanolayer-coated Co(OH)_2_ nanoparticles on a carbon sheet for SC applications, which displayed good rate capabilities and cycle durability compared with bare Co(OH)_2_ nanoparticles without polypyrrole-coated layers [25]. Although some reports have been published on this topic, it is still important to further synthesize and design unique CPs/Co(OH)_2_ NSs to accomplish higher electrochemical performances.

Very recently, highly conductive Ag nanoparticles (NPs) on the interface of microstructured BTEMs have been illustrated to supply heterostructured materials with superior electrochemical performances [25,26]. The modified interface of Ag microparticles supplies superior electrical kinetics to heterostructured materials and faster charging transporting kinetics within samples. Mainly, the heterosurfaces were comprised of BTEM and Ag, which assist in modulating the electrical constructions of BTEM and, therefore, enhance the fundamental energy storage activities. Yu et al. fabricated Ag nanoparticles decorating Ni_0.67_Co_0.33_S forest-type structures on a Ni mesh, which illustrated an area-specific capacitance that was 1.4 times better than that of the bare Ni_0.67_Co_0.33_S nanoarray samples [27]. Lan et al. published the synthesis of CuO nanowires with decorated Ag nanoparticles, which resulted in a capacitance of up to 0.44 mAh cm^−2^ at 2 mA cm^−2^ that was twice as high as that of the bare CuO nanowires (0.2 mAh cm^−2^ at 2 mA cm^−2^) [28]. In the same manner, Mahieddine et al. reported on Li_2_Co(WO_4_)_2_@Ag samples with a capacity of up to 331 mAh g^−1^ at 1 A g^−1^, which was nearly two times higher than that of a pristine Li_2_Co(WO_4_)_2_ material (175 mAh g^−1^ at 1 A g^−1^) [29]. Thus far, there have been very limited reports on Ag-doped nanoparticles used on Co(OH)_2_ materials as hybrid capacitor electrodes. Particularly, the Ag-doped particles prepared in most of these reports were instrumented either using a chemical bath deposition or light-driven approaches that demand a higher energy consumption [25,26,27,28], or through a chemical consumption route that demands costly chemicals [30]. Thus, it is reasonably advisable to develop a stable and easy approach to design Ag-doped nanoparticles for wearable Co(OH)_2_ NSs with improved energy-storing activities.

In this work, we report, for the first time, the heterogeneous interface construction of a Ag-doped@Co(OH)_2_@polypyrrole composite structure based on a Ni foam through a hydrothermal synthesis route. There are numerous advantages to the hydrothermal process, such as easy size control, uniform morphology, an excellent dispersion of the product, low cost, easy experimental setup, and a high yield. The electrochemical and physical characteristics of the Ag-doped@Co(OH)_2_@polypyrrole composite synthesized were studied. The Ag-doped@Co(OH)_2_@polypyrrole composite exhibited a superior specific capacitance of 291.2 mA h g^−1^ at a current density of 2 A g^−1^ and showed a good cycling stability after 5000 cycles at 5 A g^−1^. The results of this study show that the Ag-doped@Co(OH)_2_@polypyrrole composite can be used in next-generation flexible energy storage systems.

## 2. Experimental Section

### 2.1. Materials

All the chemicals, such as cobalt nitrate hexahydrate (Co (NO_3_)_2_. 6H_2_O), sodium p-toluenesulfonate (C_7_H_7_NaO_3_S), sodium carbonate (Na_2_CO_3_·H_2_O), pyrrole monomer, silver nitrate (AgNO_3_), polyvinylpyrrolidone (K30, (C_6_H_9_NO)_n_), and glucose (C_6_H_12_O_6_·H_2_O), were purchased from Sigma-Aldrich. The Ni foam was purchased from Japan. All the reagents were analytically graded and used without further purification.

### 2.2. Synthesis of Ag-Doped@Co(OH)_2_ Nanoparticles

The doping of Ag particles was executed using a silver mirror reaction. Before the experimental procedure, nickel foam (2 × 2 cm^2^) was carefully cleaned with a 2.0 M electrolytic KOH solution to remove and eliminate the influence of the NiOOH layer from the surface, then rinsed with deionized water and absolute ethanol for various time periods, and was, finally, dried in a vacuum oven at 70 °C. In the first step, Ag-doped@Co(OH)_2_ nanoparticles were fabricated using a facile hydrothermal route as follows: In brief, the Ni foams were put into 50 mL DI water that contained AgNO_3_ (0.04 M), glucose (0.02 M), and polyvinylpyrrolidone (3.0 g) for 14 h. The hydrothermal process was carried out at 120 °C for 4 h. After the hydrothermal process, the products were collected using centrifugation and washed with DI water several times. The Ag-doped@Co(OH)_2_ nanoparticles were then obtained after drying the products at 60 °C overnight. In the second step, for comparison, bare Co(OH)_2_ nanoparticles were also fabricated using an easier process of the hydrothermal route.

### 2.3. Preparation of Ag-Doped@Co(OH)_2_@Polypyrrole Nanosheets

In the third step, finally, the Ag-doped@Co(OH)_2_@polypyrrole NSs composite was synthesized utilizing the following detailed process: The polymerization procedure was executed in a 50 mL DI water solution that contained Na_2_CO_3_ (0.3 M), pyrrole (0.3 M), and sodium p-toluenesulfonate (0.2 M). The prepared Ag-doped@Co(OH)_2_ on Ni foam was put into a 40 mL mixed aqueous solution containing Na_2_CO_3_ (0.3 M), pyrrole (0.3 M), and sodium p-toluenesulfonate (0.2 M), and allowed to stand for 9 h in a closed blackout box. The obtained product was named Ag-doped@Co(OH)_2_@polypyrrle. The mass loading of the active material Co(OH)_2_ nanoparticles, Ag-doped@Co(OH)_2_ NPs, and Ag-doped@Co(OH)_2_@polypyrrole NS electrodes on the Ni foam substrate was calculated to be 2.1, 3.7, and 4.6 mg cm^−2^, respectively, with an analytical balance (accuracy of 0.02 mg).

### 2.4. Measurements and Characterizations

The morphological structures of the Co(OH)_2_ nanoparticles, Ag-doped@Co(OH)_2_, and Ag-doped@Co(OH)_2_@polypyrrle were perceived using field emission scanning electron microscopy (FE-SEM, JSM-7800F, Busan, Korea) and a transmission microscope (TEM, JEM-2100F, Busan, Korea). A PANalytical X’Pert PRO instrument was used to examine the crystal phase with X-ray diffraction (XRD, Bruker D8 Advance, Busan, Korea). The formation and interactions among the various components of the prepared electrode materials were studied with an X-ray photoelectron spectrometer (XPS, ESCCALAB 250Xi, Busan, Korea). In a three-electrode system with a Pt counter electrode and an Ag/AgCl reference electrode, the electrochemical performances of the produced electrodes were measured by using a Bio-Logic electrochemical workstation instrument. A potentiostat/galvanostat apparatus was used to conduct cyclic voltammetry (CV), galvanostatic charge/discharge (GC/D), and electrochemical impedance spectroscopy (EIS). At the open-circuit potential, frequency ranges from 0.05 Hz to 200 kHz were used for the EIS measurement. The specific capacity (*Q_sc_*, mAh g^−1^) values were calculated from the GCD plots in the FHSCs full device and 3-electrode configuration, using the following formula [31,32,33]:

(1) $$Q_{SC}=\frac{I\times \Delta t}{m\times 3.6}$$

where *I* (A), Δ*t* (s), and *m* (g) have their conventional meanings.

## 3. Results and Discussion

**Figure 1:**
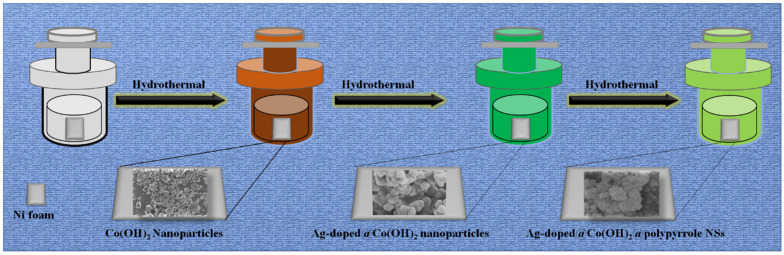
Schematic illustration of the synthesis of Ag-doped@Co(OH)_2_@polypyrrole NSs.

As a schematic illustration in Figure 1, in the first step, Ag-doped@Co(OH)_2_ nanoparticles were fabricated with a facile hydrothermal route. Particularly, Ag doping was recommended as a vital source, owing to its high capacitance data, cost-effectiveness, friendliness, and innate abundance. Moreover, the known features of Ag-doped metals can be ascribed to their high conductivity and outstanding retention due to their inherited capabilities during the charging–discharging process. Evidentially, a combination of BETMs with an Ag-doped conductive matrix-type nickel foam can enhance the cycling performance and rate capability of materials. In the second step, for comparison, bare Co(OH)_2_ nanoparticles were also fabricated using an easier process of the hydrothermal route. Afterwards, Co(OH)_2_ nanoparticles were grown through Ag-doped nanoparticles to produce the Ag-doped@Co(OH)_2_ nanoparticles using a hydrothermal process. Eventually, polypyrrole nanoparticles were decorated onto Ag-doped@Co(OH)_2_ to generate the Ag-doped@Co(OH)_2_@polypyrrole NSs. In such NSs, Ag-doped particles develop on Co(OH)_2_ nanoparticles, which greatly consume their self-aggregations. Thus, they are favored for the supply of richer active sites, powerful charge transportations, and notable mechanical stabilities. In the third step, finally, the Ag-doped@Co(OH)_2_@polypyrrole NSs composite was synthesized utilizing the following detailed process: Notably, the decoration of Ag particles on the Co(OH)_2_ nanostructures resulted in the generation of Ag-doped@Co(OH)_2_ particle interfaces, which could boost the electrical kinetics of active electrodes and also consisted of faster electron transfers at the Ag-doped@Co(OH)_2_ surface, as well as allowing for the easier adsorption of electrolyte electrons at the working/electrolyte interfaces.

**Figure 2:**
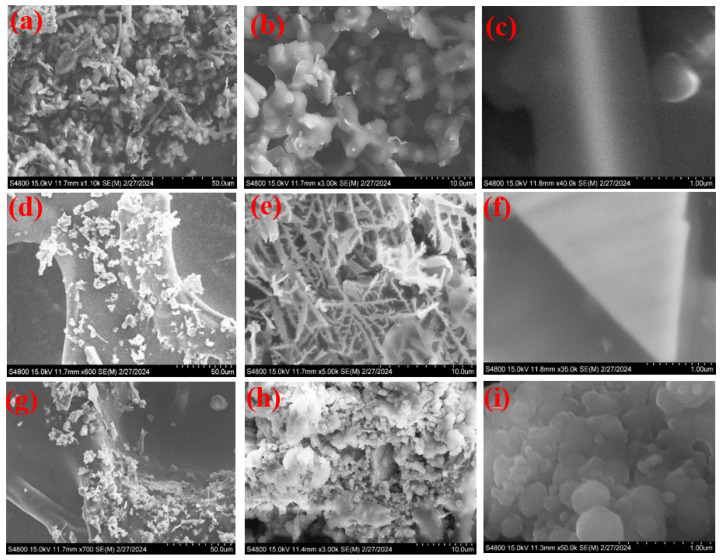
SEM images of the prepared Co(OH)_2_ nanoparticles (**a**–**c**), Ag-doped@Co(OH)_2_ nanoparticles (**d**–**f**), and Ag-doped@Co(OH)_2_@polypyrrole NSs (**g**–**i**).

Figure 2a displays lower-magnification SEM data of the bare Co(OH)_2_ nanoconstruction growing on the Ni mesh. The zoomed SEM images disclosed that these nanoparticles consisted of smooth interfaces (Figure 2b,c), and that they were morphological in length and 30–50 nm in diameter. Subsequently, the Co(OH)_2_ nanoparticles on the Ni mesh acted as skeletons to develop Co(OH)_2_ particles using the hydrothermal route. As displayed in Figure 2d,e, the SEM images of the Ag-doped@Co(OH)_2_ nanoparticles indicated a rough interface, which was due to the growth of Co(OH)_2_ nanoparticles on Ag-doped nanoparticles, as obviously displayed in Figure 2f. As illustrated, a high number of Co(OH)_2_ nanoparticles was clustered together. For these constructions, we saw that open voids appeared among the closed Co(OH)_2_ nanoparticles. As illustrated in Figure 2g–i, the SEM data show the Ag-doped@Co(OH)_2_ nanoparticles decorated with Ag particles produced utilizing a hydrothermal process. Many polypyrrole particles remained on the Co(OH)_2_ nanoparticles, revealing the successful preparation of polypyrrole nanoparticles. Furthermore, the EDS elemental mapping of Ag-doped@Co(OH)_2_@polypyrrole NSs (Figure S2a–c) demonstrated that Co, O, and Ag were homogeneously distributed in the sample.

**Figure 3:**
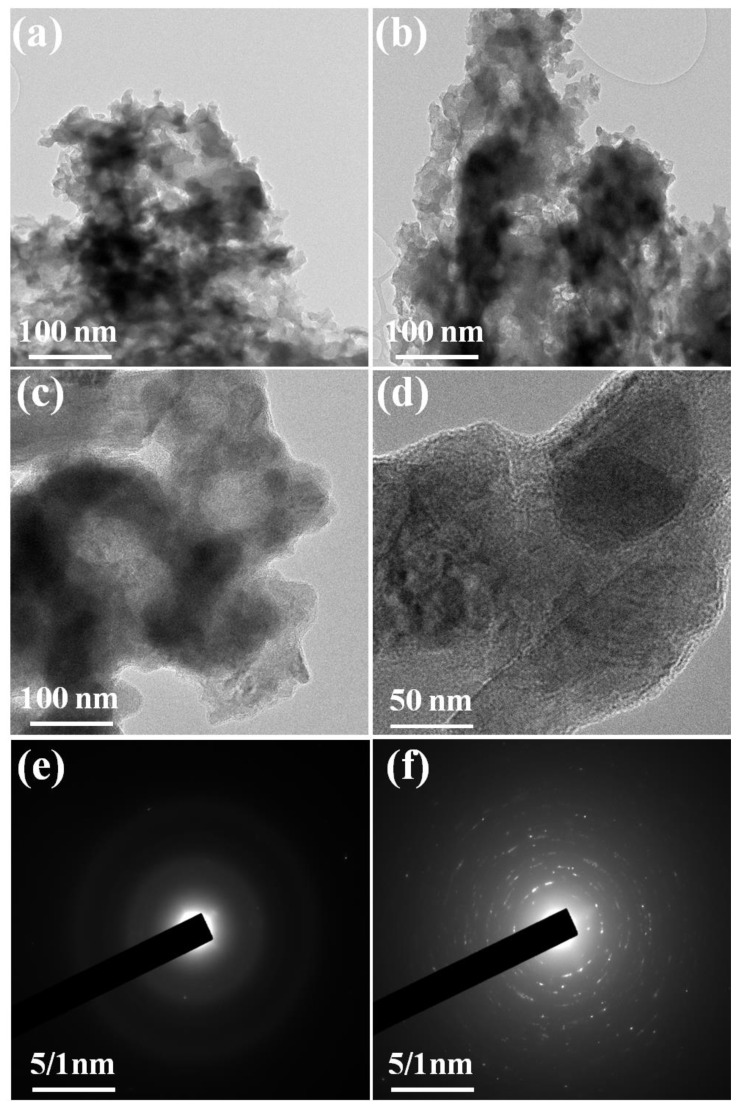
TEM images of the prepared Co(OH)_2_ nanoparticles (**a**), Ag-doped@Co(OH)_2_ NPs (**b**), and Ag-doped@Co(OH)_2_@polypyrrole NSs (**c**,**d**); SAED pattern of Ag-doped@Co(OH)_2_ NPs and Ag-doped@Co(OH)_2_@polypyrrole NSs (**e**,**f**).

The structural morphology of the prepared Co(OH)_2_ nanoparticles, Ag-doped@Co(OH)_2_ nanoparticles, and Ag-doped@Co(OH)_2_@polypyrrol NSs was examined by using an HRTEM analysis. Additionally, the recorded images are shown in Figure 3a–d. The HRTEM images revealed the presence of some agglomerated nanoparticles with tube-like structures. Figure 3a illustrates Ag-doped@Co(OH)_2_ nanoparticles with a diameter of 0.265 nm, on which tubular agglomeration was initiated. Particularly, no lattice fringe was perceived in the higher-resolution TEM image, revealing that these polypyrrole nanoparticles were advantageous due to their obvious agglomeration, as displayed in Figure 3b. Comparable with the TEM data of the Ag-doped nanoparticles, the Ag-doped@Co(OH)_2_@polypyrrole nanosheets with a thickness of 5–15 nm seemed to form the Ag-doped@Co(OH)_2_@polypyrrole NSs on the polypyrrole particles, as depicted in Figure 3c. Figure 3d displays that the Ag-doped@Co(OH)_2_@polypyrrole NSs of Ag nanoparticles were correlated on the Co(OH)_2_ particles, as well as polypyrrole nanoparticles, which matched with the Ag crystal of the (200) plane [34,35]. Most importantly, the Ag-doped@Co(OH)_2_@polypyrrole NSs of the HRTEM results matched well with the XRD results, and, also, it was noticed that the prepared working electrode possessed the Ag, Co(OH)_2_, and polypyrrole nanostructures with their phases without any adaptations. The pattern of SAED in Figure 3e,f shows clear diffraction rings that were correlated to the typical polycrystalline nature of Ag-doped@Co(OH)_2_ NPs and Ag-doped@Co(OH)_2_@polypyrrole NSs.

**Figure 4:**
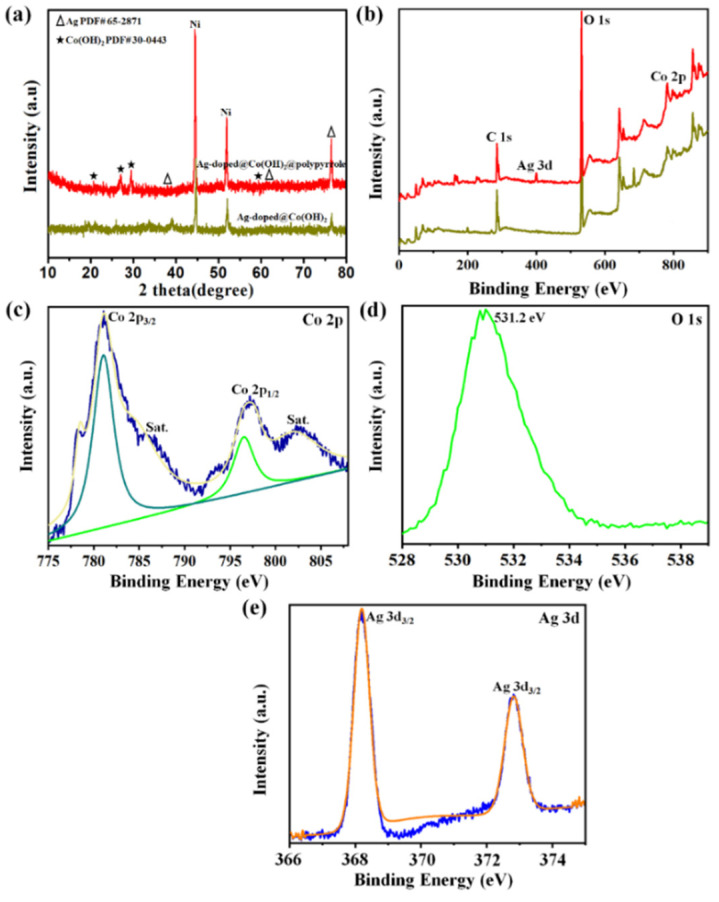
The XRD patterns for the electrode materials (**a**); the XPS survey spectrum (**b**) and the XPS spectra of Co 2p (**c**), O 1 s (**d**), and Ag 3d (**e**) of the Ag-doped@Co(OH)_2_@polypyrrole NSs.

The structure and phase purity of the prepared Ag-doped@Co(OH)_2_ NPs and Ag-doped@Co(OH)_2_@polypyrrole NSs were studied using the XRD technique, and the recorded XRD patterns are depicted in Figure 4a. From these XRD patterns, the Co(OH)_2_ nanoparticles were shown to exhibit an amorphous behavior with very poor broad peaks at 2θ = 18°–35°. Contrastingly, the individual Ag-doped@Co(OH)_2_ nanoparticles appeared to have a hexagonal crystal phase of Co(OH)_2_ (JCPDS no. 30-0443) with diffraction angles of 19.4°, 32.6°, 37.8°, 57.7°, and 61.6° [34,35]. After developing Ag-doped particles onto the polypyrrole nanoparticles, the peaks of the Co(OH)_2_ crystalline phases could still be seen in the XRD observation. After wearable polypyrrole particles were added on to Ag-doped@Co(OH)_2_, new diffraction angles showing at 38.2°, 64.7°, and 77.54° matched well with the metallic cubic crystalline of Ag (JCPDS no. 65-2871) [36], proving the generation of PPy@Co(OH)_2_@Ag NSs.

Since the Ag-doped@Co(OH)_2_@polypyrrole NSs were developed from Co(OH)_2_, Ag, and polypyrrole crystalline phases, they were expected to have a combination of characteristics from all these materials. Likewise, the absence of remaining impurities in the recorded diffractions corroborated the purity phases of the developed samples.

The XPS spectra of the Ag-doped@Co(OH)_2_ NPs and Ag-doped@Co(OH)_2_@polypyrrole NSs were then used to analyze the detailed compositions and the recorded XPS survey spectrum, along with the high-resolution spectra of individual elements, which are illustrated in Figure 4b–e. In addition, the XPS spectrum of the Co(OH)_2_ nanoparticles is displayed in Figure S1. All these spectra were optimized with the C 1s peak appearing at 284.7 eV. The existence of the Co 2p, C 1s, O 1s, and Ag 3d peaks in the survey spectrum revealed that the prepared samples were composed of Co-, Ag-, C-, and O-related compounds (Figure 4b). Co 2p_3/2_ and Co 2p_1/2_ were characterized at binding energies of 781 to 791 eV and 798.7 to 804.7 eV, respectively. The valence states of +2 and +3 were split in both Co 2p_3/2_ and Co 2p_1/2_ peaks [37,38,39]. The deconvoluted spectrum of O 1s represented three different peaks that appeared at 532.25, 530.89, and 529.19 eV [40,41,42]. Among these peaks, the peak at 532.25 was attributed to the atmospheric oxygen species. The peak at 530.89 eV was related to the surface-adsorbed hydroxyl molecules [43,44]. Moreover, the Ag 3d spectra of the Ag-doped@Co(OH)_2_@polypyrrole NSs illustrated the Ag 3d_3/2_ and Ag 3d_5/2_ angles at 374.3 and 368.3 eV, respectively (Figure 4e) [45]. This spin–orbit split of 7 eV was expressive of metallic Ag^0^, confirming the successful deposition of Ag particles [46,47,48,49], which could also be correlated to the good electronic interactions in the Ag-doped@Co(OH)_2_@polypyrrole NS sample.

### Electrochemical Properties of Electrode Materials

**Figure 5:**
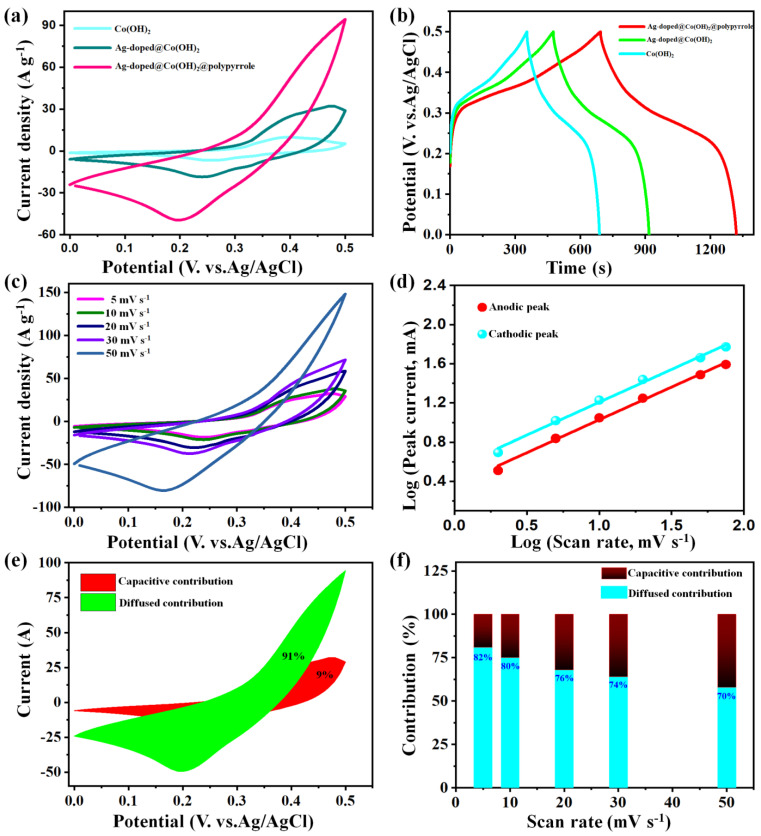
CV data at 5 mV s^−1^ (**a**) and GCD data at 2 A g^−1^ (**b**) of Co(OH)_2_ nanoparticles, Ag-doped@Co(OH)_2_ NPs, and Ag-doped@Co(OH)_2_@polypyrrole NS electrodes; CV curves (**c**) and log(*i*) vs. log(*v*) plots (**d**) of the Ag-doped@Co(OH)_2_@polypyrrole NS electrode; diffusion and capacitive contributions of the Ag-doped@Co(OH)_2_@polypyrrole NS electrode at 5 mVs^−1^ (**e**); charge storage contributions of the Ag-doped@Co(OH)_2_@polypyrrole NS electrode at various scan rates (**f**).

The charge–discharge characteristics of the as-developed Co(OH)_2_ nanoparticles, Ag-doped@Co(OH)_2_ NPs, and the heterogeneous interface of the Ag-doped@Co(OH)_2_@polypyrrole NSs were initially executed via a three-electrode configuration to synthesize the electrochemical performance (Figure 5a). Particularly, the CV plots at 5 mV s^−1^ were conducted to reveal the potential window due to a high scanning rate that would have been hiding the water electrolysis reaction. It could be observed that all these samples illustrated vital redox peaks, revealing a battery-type nature [50,51].

The possible redox mechanism of the working electrodes under pure and redox-additive electrolytes is given in Equations (2) and (3) [51,52,53]:

Co(OH)_2_ + OH^−^
↔ CoOOH + H_2_O + e^−^ (2)

CoOOH + OH^−^
↔ CoO_2_ + H_2_O + e^−^ (3)

The Ag-doped@Co(OH)_2_@polypyrrole NS electrode showed greater CF values than the Co(OH)_2_ nanoparticles and Ag-doped@Co(OH)_2_ NP samples composed of a single component, revealing the synergistic effects of polypyrrole, Ag, and Co(OH)_2_ in the Ag-doped@Co(OH)_2_@polypyrrole NSs. These synergistic effects could provide numerous redox reactions for charge storage and better surface properties with a larger surface area and pore volume. Thus, the above advantages indicated that the impact of the heterogeneous interface of the Ag-doped@Co(OH)_2_@polypyrrole NS composite achieved a boost in the SC’s performance and an impressive electrochemical performance with a high specific capacity when compared to the remaining active material of the Co(OH)_2_ nanoparticles and Ag-doped@Co(OH)_2_ NPs. Moreover, the GCD data of the Co(OH)_2_ nanoparticles, Ag-doped@Co(OH)_2_ NPs, and Ag-doped@Co(OH)_2_@polypyrrole NS electrodes are shown in Figure 5b to be at 2 A g^−1^. The smaller IR drop in the GCD plot was attained for the Ag-doped@Co(OH)_2_@polypyrrole NS electrode due to its favorable morphology, with regular aggregations comparable with the Co(OH)_2_ nanoparticles and Ag-doped@Co(OH)_2_ NP electrodes. The GCD curves of the Ag-doped@Co(OH)_2_@polypyrrole NS sample phenomena were consistent with those observed in the CV curves, which facilitated an improvement in the electrochemical properties of this composite. Thus, the above advantages indicated that the impact of the active material of the heterogeneous interface of the Ag-doped@Co(OH)_2_@polypyrrole NS composite achieved impressive electrochemical performance and boosted the SC’s performance, with a high specific capacity when compared to the remaining Co(OH)_2_ nanoparticles and Ag-doped@Co(OH)_2_ NP active materials.

Figure 5c demonstrates the CV plots of the Ag-doped@Co(OH)_2_@polypyrrole NS electrode at various scanning rates of 5–50 mVs^−1^. Their configurations of redox peaks were attained with increases in the scanning values, indicating a desirable energy storage reversibility. The CV tests of other Co(OH)_2_ nanoparticles and Ag-doped@Co(OH)_2_ NP samples also displayed the same behavior. Figure 5d depicts the linear curves of log (*i*, peak current) vs. log (*v*, scan rate) from the Ag-doped@Co(OH)_2_@polypyrrole NS sample. These linear correlations obeyed the regular equations of *i* = *a v^b^* (*a* and *b*—values indicate an adjustable parameter and the slope of the Log*i* vs. Log*v* plot) [46,50]. Accordingly, the correlating *b*-values were measured to be both 0.54, revealing a diffusion-controlled battery-type nature at the Ag-doped@Co(OH)_2_@polypyrrole NS sample. Moreover, the capacitive contribution and quantified controlled diffusion to the total volume of this sample could be obtained from this equation: *i* = *k_1_v* + *k_2_v*^1/2^ [46,50], in which *k_1_v* and *k_2_v*^1/2^ were attributed to the capacitance and diffusion-controlled correlations, respectively. A 91% diffusion-controlled contribution was delivered for the Ag-doped@Co(OH)_2_@polypyrrole NS electrode at 5 mV s^−1^ (Figure 5e), and this result decreased to 71% at a high 30 mV s^−1^ (Figure 5f), confirming the diffusion-controlled charging stored of the sample electrode.

**Figure 6:**
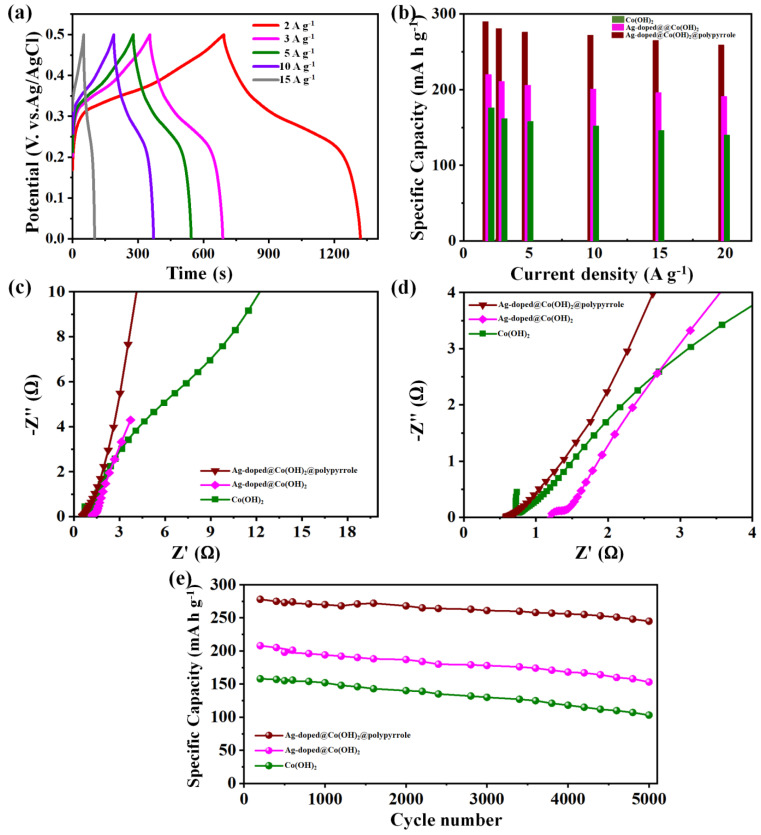
GCD curves of the Ag-doped@Co(OH)_2_@polypyrrole NS electrode at different current densities (**a**); specific capacitance at various current densities (**b**), Nyquist plots (**c**), and zoomed Nyquist plots (**d**), and cyclic stability (**e**) of the pristine Co(OH)_2_, Ag-doped@Co(OH)_2_ NPs, and Ag-doped@Co(OH)_2_@polypyrrole NS electrodes.

The GCD plots of the Ag-doped@Co(OH)_2_@polypyrrole NS electrode were obtained at varied current densities of 2–15 A g^−1^ (Figure 6a). The measured specific capacities of various electrode materials as a function of current density value were plotted in Figure 6b. The Co(OH)_2_ nanoparticles, Ag-doped@Co(OH)_2_ NPs, and Ag-doped@Co(OH)_2_@polypyrrole NS samples delivered specific capacities of 176, 220.3, and 291.2 mAhg^−1^ at 2 A g^−1^, respectively. Most importantly, at 15 A g^−1^, the Ag-doped@Co(OH)_2_@polypyrrole NS electrode remained at 84% of its capacity at a 2 A g^−1^ current density. These Ag-doped@Co(OH)_2_@polypyrrole NS capacity retentions were greater than those of the Ag-doped@Co(OH)_2_ NPs (73%) and Co(OH)_2_ nanoparticles (68%), which facilitated an improvement in the electrochemical properties of this composite.

Electrochemical impedance spectroscopy (EIS) was executed to find out the frequency-dependent charge transport among the electrode surfaces and electrolytes for the Co(OH)_2_ nanoparticles, Ag-doped@Co(OH)_2_ NPs, and Ag-doped@Co(OH)_2_@polypyrrole NSs. The recorded EIS spectra (Figure 6c,d) confirmed that the electrochemical performance of the fabricated working electrodes was affected by two various resistances, such as the solution resistance (*R*_S_) and contact resistance (*R*_CT_). The EIS plots of various samples electrode, including the Co(OH)_2_ nanoparticles, Ag-doped@Co(OH)_2_ NPs, and Ag-doped@Co(OH)_2_@polypyrrole NS electrodes exhibited small internal resistances (*R*s) of 1.75, 1.24, and 0.75 Ω, respectively. Concurrently, they also displayed low charging transfer resistances (*R*ct) of 3.52, 2.72, and 2.15, respectively. Moreover, the Ag-doped@Co(OH)_2_@polypyrrole NS sample illustrated a straighter vertical lining than the remaining samples in the lower-frequency area, explaining the low Warburg impedance that was suggestive of the rapid ion diffusion of electrolytes within sample electrodes [46,50,51,52,53]. Thus, the low *R*ct, *R*s, and low Warburg impedance results for the Ag-doped@Co(OH)_2_@polypyrrole NS electrodes corroborated the ultra-electrochemical performance and rapid charging transport kinetics. The lower *R*_CT_ and a shift to linearity in the low-frequency area enabled a rapid electron transfer throughout charging–discharging. Thus, the Ag-doped@Co(OH)_2_@polypyrrole NS electrode revealed a higher specific capacitance compared to those of the other electrodes.

The cyclic stability under extreme load is another crucial parameter of the electrode material to be applied in supercapacitor applications. The cyclic stability for each sample of Co(OH)_2_ nanoparticles, Ag-doped@Co(OH)_2_ NPs, and Ag-doped@Co(OH)_2_@polypyrrole NS electrodes was also measured by repeating the GCD plots 5000 times at 5A g^−1^ (Figure 6e). The Ag-doped@Co(OH)_2_@polypyrrole NS electrode achieved a higher initial capacity retention of 86% than the Ag-doped@Co(OH)_2_ NP (81%) and Co(OH)_2_ nanoparticle (74%) samples after 5000 cycles (Figure 6e). The specific capacity results were compared with other recently published results, displayed in Table 1. From the table, we could see the specific capacity of the as-developed Ag-doped@Co(OH)_2_@polypyrrole NSs was higher than the other Co(OH)_2_ with doped materials. The reason why the Ag-doped@Co(OH)_2_@polypyrrole NSs achieved a better electrochemical performance may be due to the mesoporous nanostructure and the synergistic effect of the Ag-doping, polypyrrole, and Co(OH)_2_.

## 4. Conclusions

In summary, a simple hydrothermal synthesis method was employed to successfully synthesize the heterogeneous interface of Ag-doped@Co(OH)_2_@polypyrrole NSs, and their structural and electrochemical properties were investigated. Initially, the Co(OH)_2_ nanoparticles were developed onto polypyrrole with the assistance of Ag-doped particles to generate the Ag-doped@Co(OH)_2_@polypyrrole NSs that boosted the total number of electroactive sites and enhanced the effective charging transportations with a good structural stability. As a result, the Ag-doped@Co(OH)_2_@polypyrrole NS material achieved better energy storage activities than the remaining electrodes, which was attributed to the interfacial electronic connections among the Ag nanoparticles, Co(OH)_2_ particles, and polypyrrole. The Ag-doped@Co(OH)_2_@polypyrrole composite exhibited a high specific capacity of 291.2 mAh g^−1^ at a current density of 2 A g^−1^, and showed a good cycling stability after 5000 cycles at 5 A g^−1^. This work could provide a very convenient approach to improve the charging storage performance of Co(OH)_2_-based battery-type materials for supercapacitors.

## Figures and Tables

**Table 1:** Comparison of specific capacity values of recently published metal oxides with multicomponent-based electrodes with our highly efficient Ag-doped@Co(OH)_2_@polypyrrole NS electrode in 3-electrode configuration.

Electrode	Fabrication Method	Electrolyte	Capacitance (Current Density)	Cycling Stability (no. of Cycles)	Ref.
Co(OH)*_x_*CO_3_ with hierarchical flowery architecture	Hydrothermal	2 M KOH	550 F g^−1^ (2 Ag^−1^)	99.5% (1500)	[54]
Flower-like Co(OH)_2_/N-doped graphene composite	Hydrothermal	3 M KOH	2276 F g^−1^ (1 Ag^−1^)	93.5% (2000)	[55]
Delaminated α-Co(OH)_2_@graphene	Hydrothermal and calcination	2 M KOH	567 F g^−1^ (1 Ag^−1^)	82% (2000)	[56]
Co(OH)_2_/graphene/Ni foam nanoelectrodes	Hydrothermal	6 M KOH	694 F g^−1^ (2 Ag^−1^)	91.9% (3000)	[57]
CNT-wrapped Co(OH)_2_ flakes	Hydrothermal-Electrodeposition	2 M KOH	603 F g^−1^ (1 mV s^−1^)	96% (1000)	[58]
Ni–Co hydroxide/CNT composites	One-step hydrothermal	2 M KOH	1151 F g^−1^ (1 Ag^−1^)	77% (10000)	[59]
Heterogeneous Co_3_O_4_-nanocube/Co(OH)_2_-nanosheet hybrid	Hydrothermal-thermal annealing	6 M KOH	1164 F g^−1^ (1.2 Ag^−1^)	97.4% (6000)	[60]
Layered α-Co(OH)_2_ nanocones	Hydrothermal reaction	3 M KOH	1055 F g^−1^ (1 Ag^−1^)	95% (2000)	[61]
Co(OH)_2_@FeCo_2_O_4_	Hydrothermal	6 M KOH	1173.43 F g^−1^ (1 Ag^−1)^	95.4% (5000)	[62]
Ag-doped@Co(OH)_2_@polypyrrole NSs	Hydrothermal	2 M KOH	291.2 mAh g^−1^ mA h g^−1^ or 1734.3 F g^−1^ (2 A g^−1^)	84% (5000)	This work

## Data Availability

No new data were created or analyzed in this study. Data sharing is not applicable to this article.

## Supplementary Materials

The following supporting information can be downloaded at: https://www.mdpi.com/article/10.3390/nano12223982/s1, Figure S1. XPS survey spectrum of pure Co(OH)_2_ nanoparticles electrode grown on Ni foam; Figure S2. EDS elemental distribution mapping of Ag-doped@Co(OH)_2_@polypyrrole NSs grown on Ni foam.
