# Correction: Arbi et al. Polypyrrole-Assisted Ag Doping Strategy to Boost Co(OH)_2_ Nanosheets on Ni Foam as a Novel Electrode for High-Performance Hybrid Supercapacitors. *Nanomaterials* 2022, *12*, 3982

**DOI:** 10.3390/nano14221829

**Published:** 2024-11-15

**Authors:** Hammad Mueen Arbi, Anuja A. Yadav, Yedluri Anil Kumar, Md Moniruzzaman, Salem Alzahmi, Ihab M. Obaidat

**Affiliations:** 1 Department of Physics, United Arab Emirates University, Al Ain 15551, United Arab Emirates; 201990023@uaeu.ac.ae (H.M.A.); yedluri.anil@gmail.com (Y.A.K.); 2 Department of Automotive Engineering, Yeungnam University, 280 Daehak-ro, Gyeongsan 38541, Gyeongbuk, Korea; anujayadav5@yu.ac.kr; 3 National Water and Energy Center, United Arab Emirates University, Al Ain 15551, United Arab Emirates; 4 Department of Chemical and Biological Engineering, Gachon University, 1342 Seongnam-daero, Seongnam-si 13120, Gyeonggi-do, Korea; mani57chem@gachon.ac.kr; 5 Department of Chemical & Petroleum Engineering, United Arab Emirates University, Al Ain 15551, United Arab Emirates

## Error in Figures

In the original publication [1], there were mistakes in the Graphical Abstract, Figures 1 and 2, as published. The authors added the wrong SEM images to Figure 2 and, as a consequence, the SME images in Figure 1 and the Graphical Abstract were also incorrect. The corrected Graphical Abstract, Figure 1 and Figure 2 appear below.


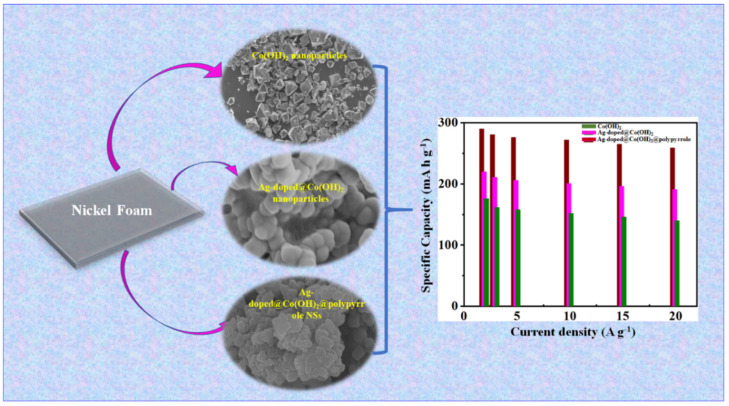


Graphical Abstract

**Figure 1:**
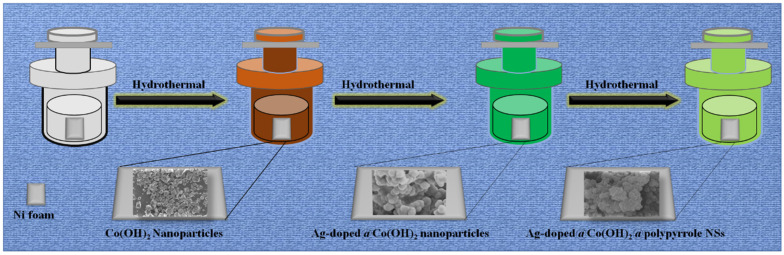
Schematic illustration of the synthesis of Ag-doped@Co(OH)_2_@polypyrrole NSs.

**Figure 2:**
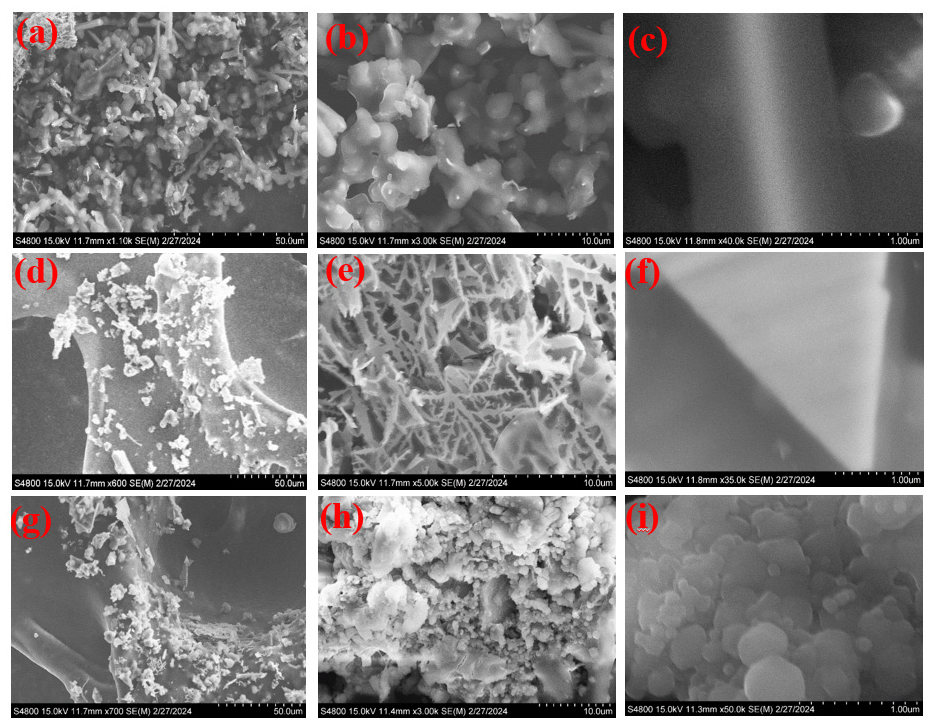
SEM images of the prepared Co(OH)_2_ nanoparticles (**a**–**c**), Ag-doped@Co(OH)_2_ nanoparticles (**d**–**f**), and Ag-doped@Co(OH)_2_@polypyrrole NSs (**g**–**i**).

In the original publication [1], there were mistakes in Figure 4. The XRD patterns were erroneously added to Figure 4. The corrected Figure 4 appears below.

## Text Correction

There was an error in the original publication [1]. Following the above error in Figure 4, a correction has been made to Section 3, Results and Discussion, Paragraph 4.

“The structure and phase purity of the prepared Ag-doped@Co(OH)_2_ NPs and Ag-doped@Co(OH)_2_@polypyrrole NSs were studied using the XRD technique, and the recorded XRD patterns are depicted in Figure 4a. From these XRD patterns, the Co(OH)_2_ nanoparticles were shown to exhibit an amorphous behavior with very poor broad peaks at 2θ = 18°–35°. Contrastingly, the individual Ag-doped@Co(OH)_2_ nanoparticles appeared to have a hexagonal crystal phase of Co(OH)_2_ (JCPDS no. 30-0443) with diffraction angles of 19.4°, 32.6°, 37.8°, 57.7°, and 61.6° [34,35]. After developing Ag-doped particles onto the polypyrrole nanoparticles, the peaks of the Co(OH)_2_ crystalline phases could still be seen in the XRD observation. After wearable polypyrrole particles were added on to Ag-doped@Co(OH)_2_, new diffraction angles showing at 38.2°, 64.7°, and 77.54° matched well with the metallic cubic crystalline of Ag (JCPDS no. 65-2871) [36], proving the generation of PPy@Co(OH)_2_@Ag NSs.”

The authors state that the scientific conclusions are unaffected. This correction was approved by the Academic Editor. The original publication has also been updated.

## Figures and Tables

**Figure 4:**
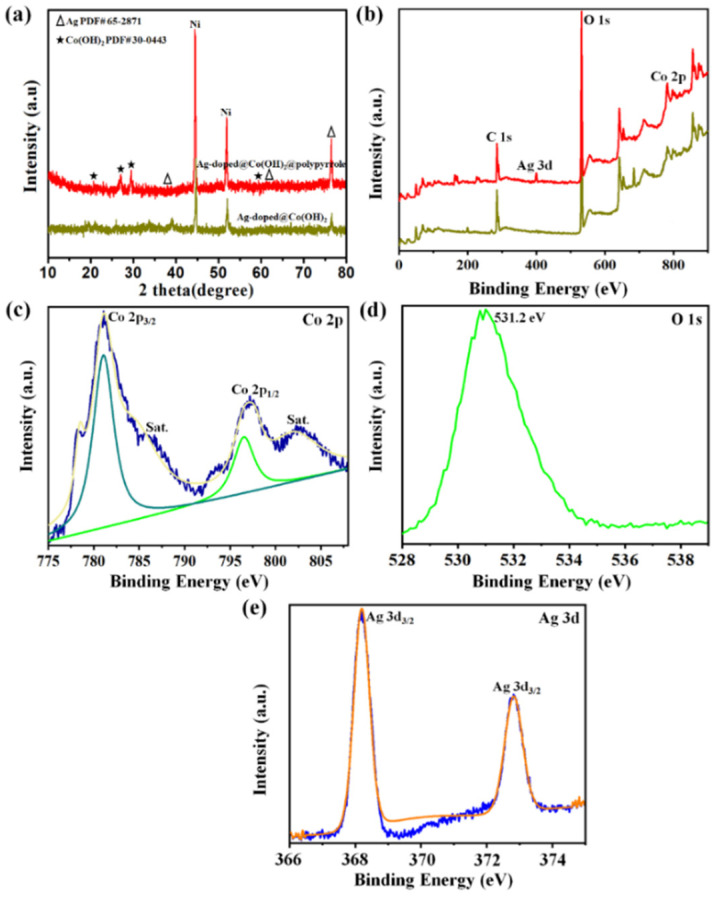
The XRD patterns for the electrode materials (**a**); the XPS survey spectrum (**b**) and the XPS spectra of Co 2p (**c**), O 1 s (**d**), and Ag 3d (**e**) of the Ag-doped@Co(OH)_2_@polypyrrole NSs.
