# Supplementary material for: Polypyrrole-Assisted Ag Doping Strategy to Boost Co(OH)2 Nanosheets on Ni Foam as a Novel Electrode for High-Performance Hybrid Supercapacitors
Source: Nanomaterials (Basel). 2022 Nov 11;12(22):3982. doi: 10.3390/nano12223982 (PMC9697904; doi:10.3390/nano12223982)
Supplement: Supplementary file 1 [file nanomaterials-12-03982-s001.zip › nanomaterials-1992453-supplementary.pdf]

<u>**Supplementary Materials**</u>

Polypyrrole-Assisted Ag Doping Strategy to Boost Co(OH)2 Nanosheets on Ni Foam as a Novel Electrode for High-Performance Hybrid Supercapacitors

Hammad Mueen Arbi [1,†], Anuja A. Yadav [2,†], Yedluri Anil Kumar [1,3], Md Moniruzzaman [4], Salem Alzahmi [3,5,*] and Ihab M. Obaidat [1,3,*]

1 Department of Physics, United Arab Emirates University, Al Ain 15551, United Arab Emirates
2 Department of Automotive Engineering, Yeungnam University, 280 Daehak-ro, Gyeongsan 38541, Gyeongbuk, Korea
3 National Water and Energy Center, United Arab Emirates University, Al Ain 15551, United Arab Emirates
4 Department of Chemical and Biological Engineering, Gachon University, 1342 Seongnam-daero, Seongnam-si 13120, Gyeonggi-do, Korea
5 Department of Chemical & Petroleum Engineering, United Arab Emirates University, Al Ain 15551, United Arab Emirates
* Correspondence: s.alzahmi@uaeu.ac.ae (S.A.); iobaidat@uaeu.ac.ae (I.M.O.)
† These authors contributed equally to this work.

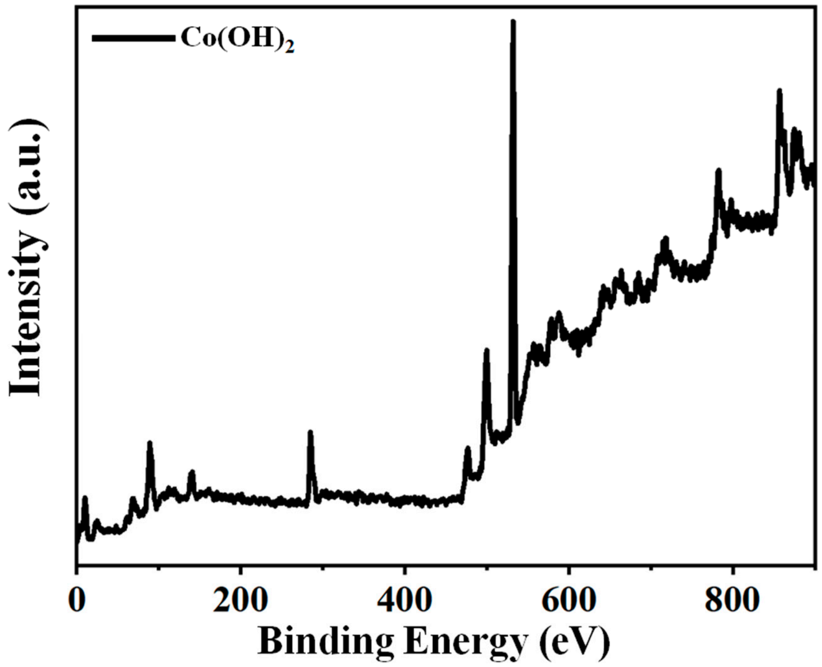

Fig. S1 XPS survey spectrum of pure $Co(OH)_2$ nanoparticles electrode grown on Ni foam.

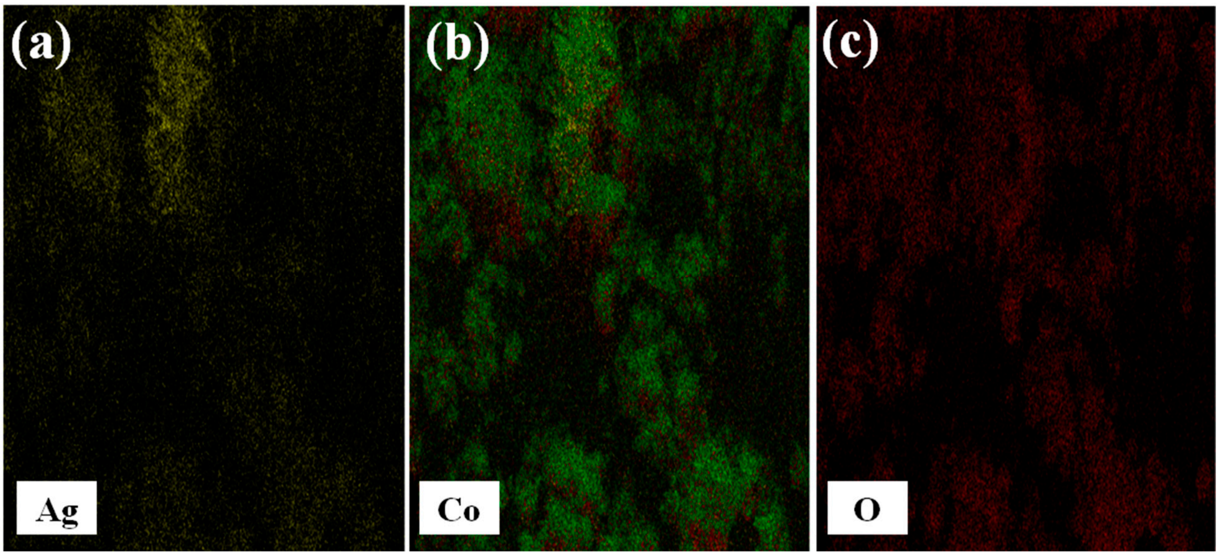

Fig. S2 (**a–c**) EDS elemental distribution mapping of Ag-doped@Co(OH)2@polypyrrole NSs grown on Ni foam.
